# Supplementary material for: Development and evaluation of a deep neural network for histologic classification of renal cell carcinoma on biopsy and surgical resection slides
Source: Sci Rep. 2021 Mar 29;11:7080. doi: 10.1038/s41598-021-86540-4 (PMC8007643; doi:10.1038/s41598-021-86540-4)
Supplement: Supplementary file 1 — Supplementary Information. [file 41598_2021_86540_MOESM1_ESM.docx]

**Development and Evaluation of a Deep Neural Network for Histologic Classification of Renal Cell Carcinoma on Biopsy and Surgical Resection Slides**

Authors: Mengdan Zhu, MS^1^, Bing Ren, MD^2^, Ryland Richards, MD^2^, Matthew Suriawinata^1^, Naofumi Tomita, MS^1^, Saeed Hassanpour, PhD^1,3,4*^

^1^Department of Biomedical Data Science, Geisel School of Medicine at Dartmouth, Hanover, NH 03755, USA

^2^Department of Pathology and Laboratory Medicine, Dartmouth-Hitchcock Medical Center, Lebanon, NH 03756, USA

^3^Department of Computer Science, Dartmouth College, Hanover, NH 03755, USA

^4^Department of Epidemiology, Geisel School of Medicine at Dartmouth, Hanover, NH 03755, USA

^*^ Corresponding Author: Saeed Hassanpour, PhD

Postal address: One Medical Center Drive, HB 7261, Lebanon, NH 03756, USA

Telephone: (603) 650-1983

Email: Saeed.Hassanpour@dartmouth.edu

**Supplementary Material**

**Table of Contents:**

**Appendix A: Inclusion and Exclusion Criteria for DHMC Data Collection**

**Appendix B: The Details of Grid Search Process**

**Figure S1: Typical Examples of Visualized Slides using the Deep Learning Model**

**Figure S2: Error Analysis**

**Figure S3: Samples of WSI Misclassification**

**Figure S4: Grad-CAM Visualization**

**Table S1: The Distribution of the Patch-Level Development Dataset**

**Table S2: Model Performance on the Patch-Level Development Set**

**Table S3: Model Performance on the TCGA Test Set Stratified by Grade**

**Appendix A: Inclusion and Exclusion Criteria for DHMC Data Collection**

Of 636 renal resection specimens available from 2015 to 2019 at DHMC, specimens were excluded if the total number of available cases in their corresponding class was less than 20 cases (e.g., renal pelvic urothelial carcinoma). In total, 486 specimens met the criteria and were included for further analysis in our study.

The same criteria were applied to 122 RCC biopsy specimens collected from 2015 to 2017 at DHMC, and 79 specimens were included for further analysis.

**Appendix B: The Details of Grid Search Process**

We developed our whole-slide inference by exploring two parameters using a grid search. We first identified an optimal cut-off value for confidence scores of patch classification, ranging from 0.3 to 0.9 with 0.1 step size, to exclude ambiguous patch-level predictions to remove noise and improve our patch classifier's specificity. Once we found the threshold value of 0.9 is optimal in this stage, we processed each slide in the development set and pooled patch-wise predictions after confidence-filtering for each slide. Given the observed class distribution of patches for each slide and informed by the pathologists' decision-making process in clinical practice, we developed a thresholding rule to distinguish abnormal slides from normal slides. We used a grid search, ranging from 1.0% to 20.0% with 1.0% step size, on the development set, and found that an abnormal class should be present in at least 5.0% of the pooled predictions to affect the whole-slide level label. In that case, the most prevalent subtype in a slide is assigned as the slide's label. At the test time, we applied the thresholding rules using these two parameters from our grid search in the development process.


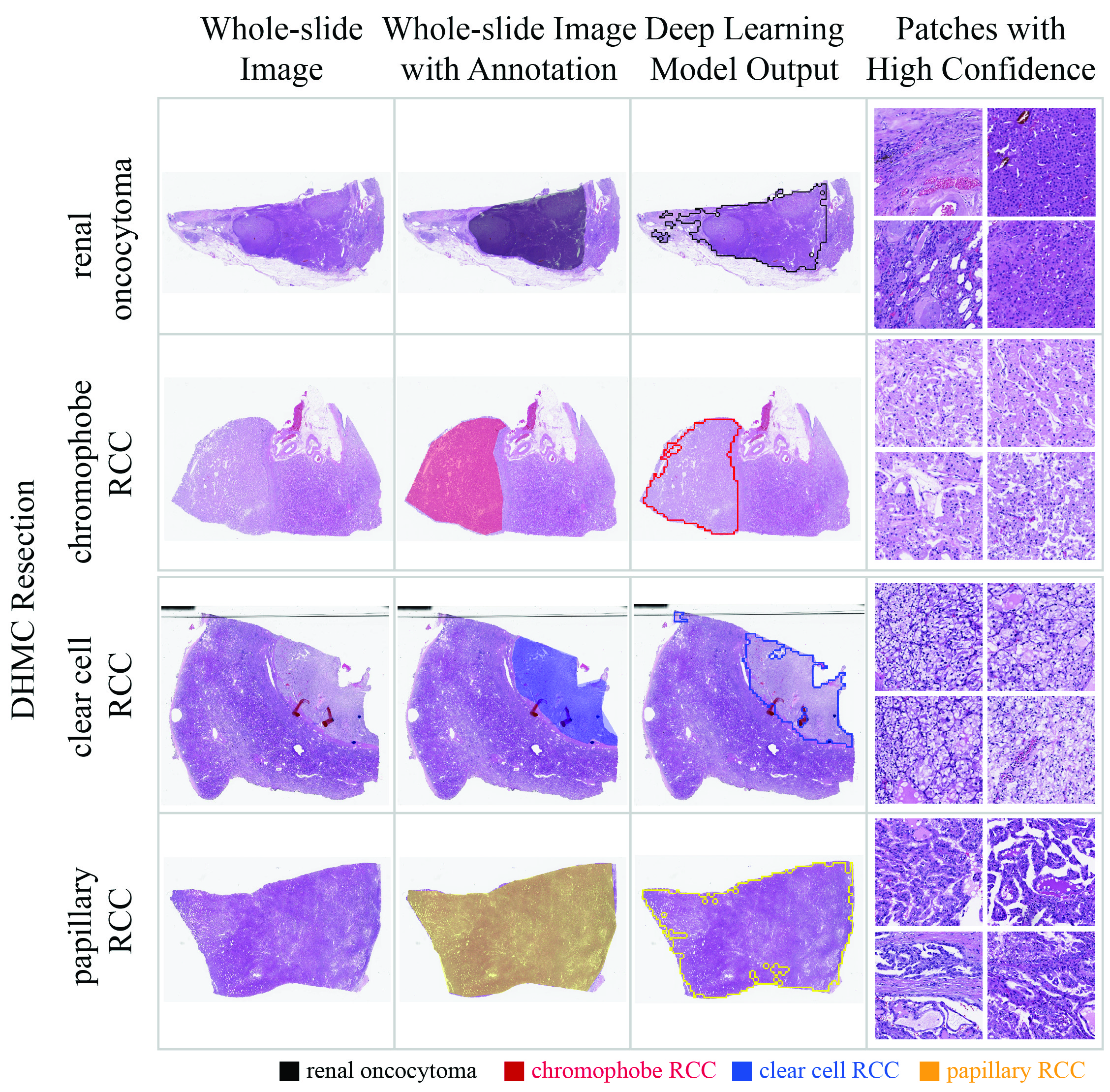


**Figure S1: Typical Examples of Visualized Slides using the Deep Learning Model.** Examples of visualized slides in our DHMC test set with highlighted regions for predicted classes. Each example slide for a subtype is presented with pathologists' annotations of abnormal areas, model output, and patches with the model's high confidence.

**
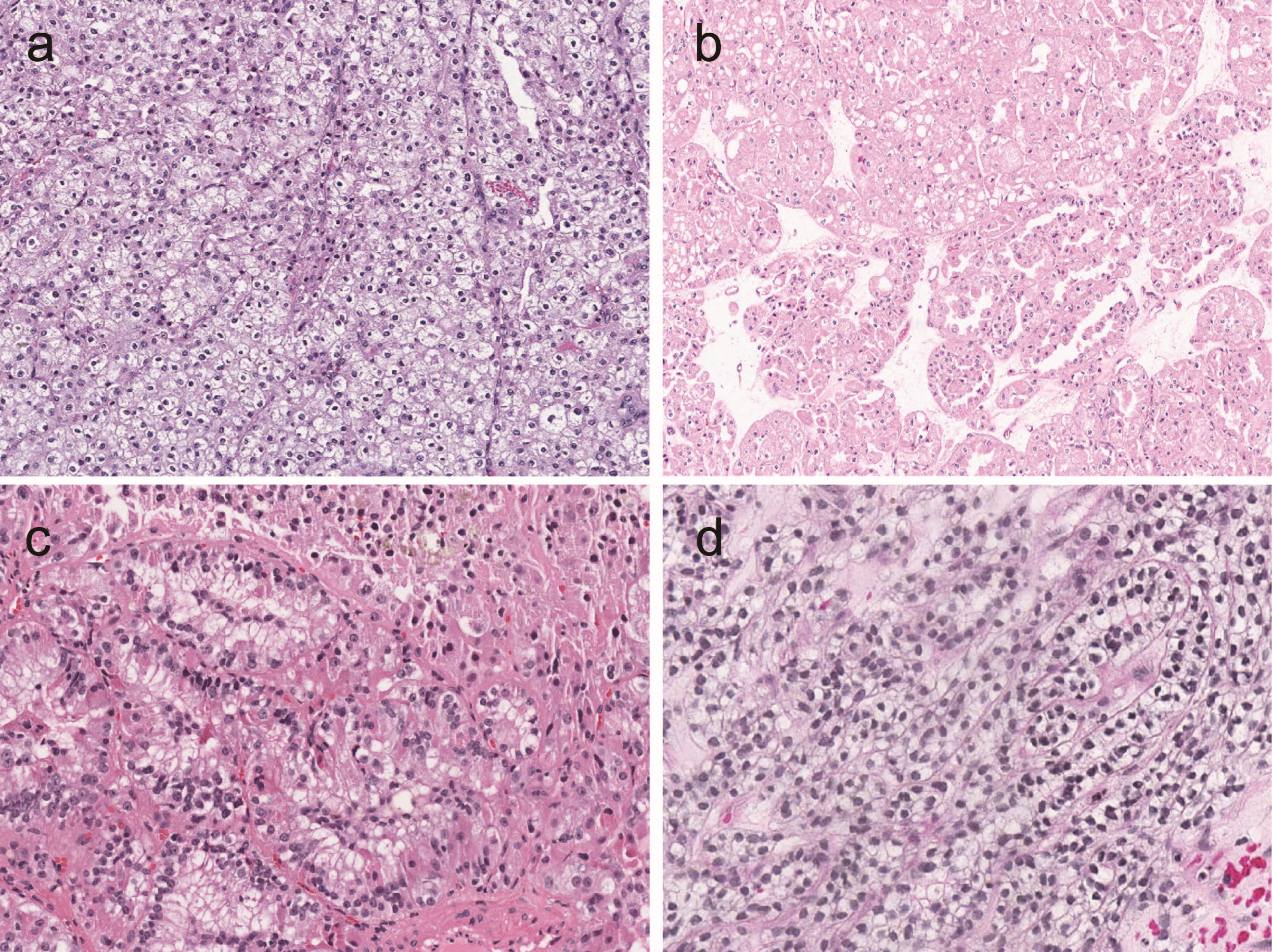
**

**Figure S2: Error Analysis. (a)** The misclassification of chromophobe RCC as clear cell RCC or papillary RCC because of the substantial clear cytoplasm or papillary structure with fibrovascular cores. **(b)** The misclassification of oncocytoma as chromophobe RCC due to focal tubular growth pattern and less characteristic stroma present. **(c)** The misclassification of papillary RCC as clear cell RCC due to focal tumor cell with clear cytoplasm and thin-walled vasculature. **(d)** The misclassification of clear cell RCC as papillary RCC due to the mimic of focal papillary formation and less clear cytoplasm.

**
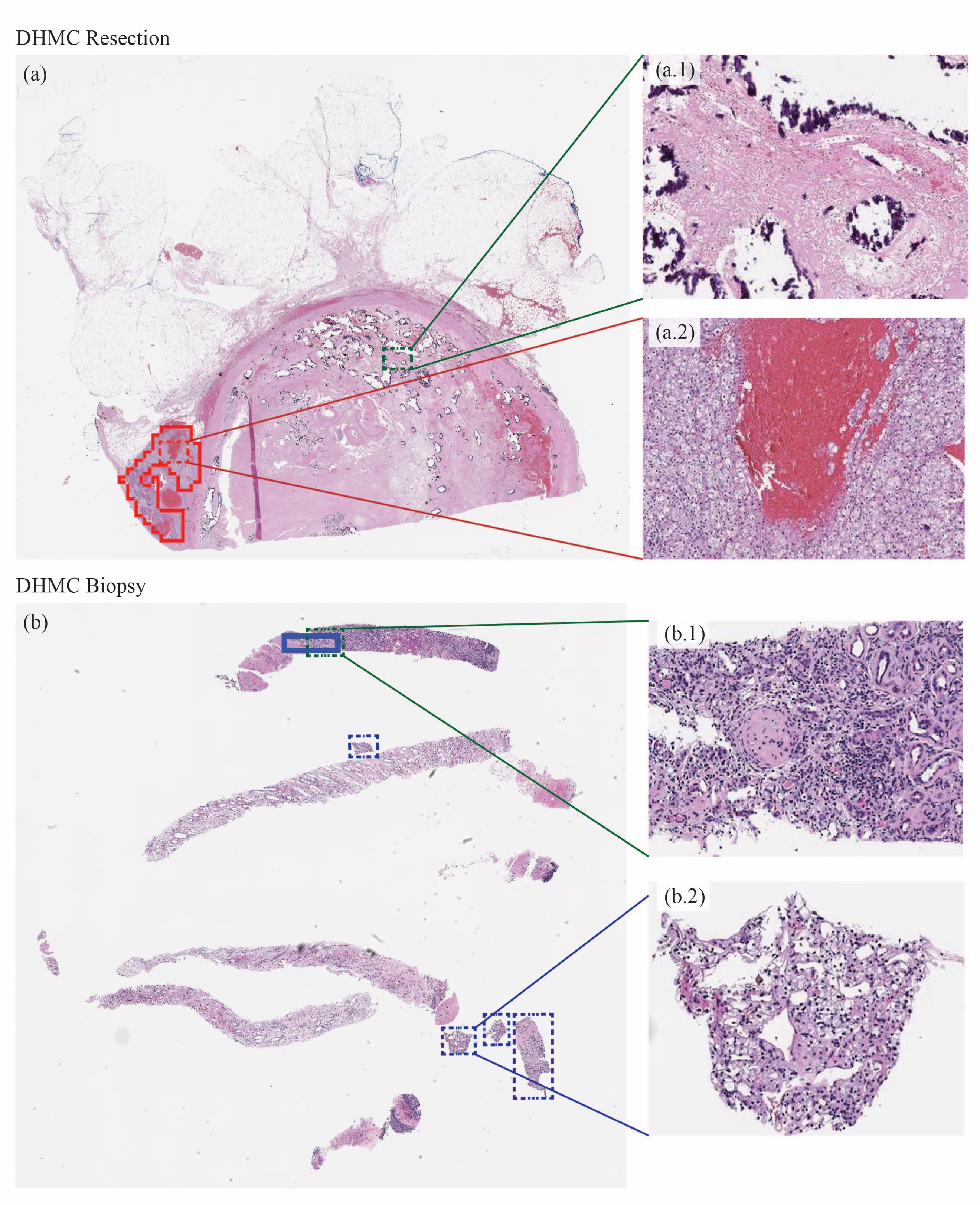
**

**Figure S3: Samples of WSI Misclassification.** (a) A chRCC sample slide from the DHMC resection test set is incorrectly identified as pRCC by our model. (b) A ccRCC sample slide from the DHMC biopsy test set that is predicted as normal. Rectangles with solid lines are the model's predictions. Boxes with dashed lines are the regions of interest and magnified on the right. In the resection case (Figure S3a), the majority area of the tissue is tumor necrosis mixed with fibrin deposition and scattered calcification (Figure S3a.1), and the viable tumor cells occupy a relatively minor portion of the tissue. The reason for failure in this case might be due to the model mistakenly recognizing the scattered calcification in a background of necrotic tissue as pRCC and failing to recognize the true viable chRCC area. In the biopsy case, the large tissue fragments are benign renal parenchyma, while the tumor cells are present in the detached small tissue core fragments. Similarly, the model mistakenly recognized the benign renal parenchyma with chronic inflammation as a tumor area and failed to recognize the true tumor cells in the small tissue fragments.

**
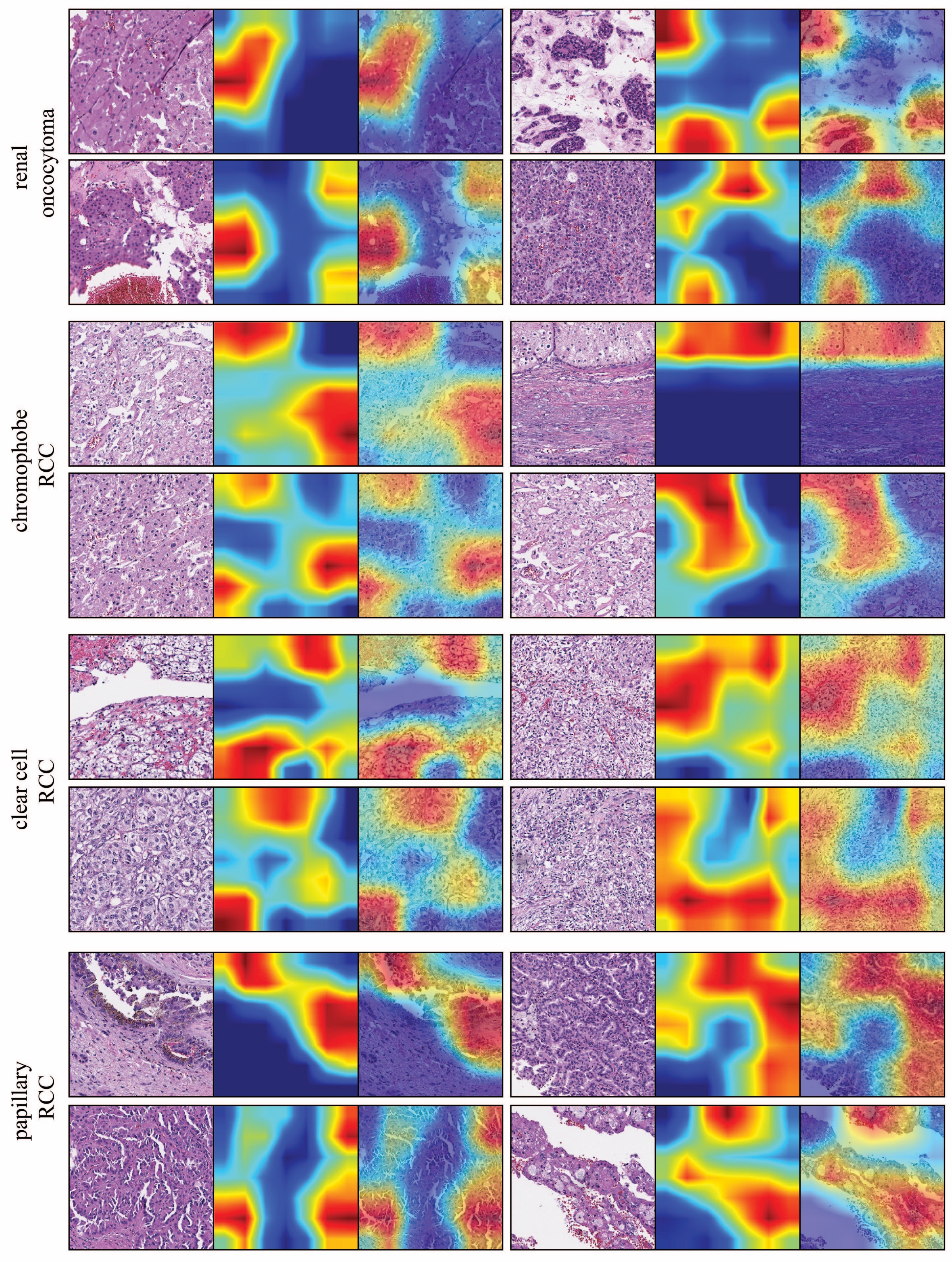
**

**Figure S4: Grad-CAM Visualization.** Examples of visualized patches in our DHMC test set with highlighted regions for predicted classes in red using the Grad-CAM technique, which utilizes the patch classification model's gradient information flow to localize the decisive features and regions in a patch. For each sample, images are original patches, raw Grad-CAM heatmaps, and superimposed heatmaps on original images.

**Table S1: The Distribution of the Patch-Level Development Dataset**

| Subtype | WSI | Patches |
| --- | --- | --- |
| normal | 5 | 7,639 |
| renal oncocytoma | 3 | 5,478 |
| chromophobe RCC | 5 | 12,308 |
| clear cell RCC | 5 | 10,989 |
| papillary RCC | 5 | 8,385 |
| Total | 23 | 44,799 |

**Table S2:** **Model Performance on the Patch-Level Development Set**

| Subtype | Precision | Recall | F1-score |
| --- | --- | --- | --- |
| normal | 1.00 | 1.00 | 1.00 |
| renal oncocytoma | 1.00 | 1.00 | 1.00 |
| chromophobe RCC | 1.00 | 1.00 | 1.00 |
| clear cell RCC | 0.83 | 1.00 | 0.91 |
| papillary RCC | 1.00 | 0.80 | 0.89 |
| Average | 0.97 | 0.96 | 0.96 |

**Table S3: Model Performance on the TCGA Test Set Stratified by Grade**

| Grade | Recall | F1-score |
| --- | --- | --- |
| G1 | 0.79 (0.71-0.83) | 0.87 (0.83-0.91) |
| G2 | 0.91 (0.86-0.95) | 0.95 (0.92-0.97) |
| G3 | 0.86 (0.82-0.91) | 0.93 (0.90-0.95) |
| G4 | 0.80 (0.74-0.85) | 0.87 (0.85-0.92) |
